# Aberrant uncertainty processing is linked to psychotic-like experiences, autistic traits, and is reflected in pupil dilation during probabilistic learning

**DOI:** 10.3758/s13415-023-01088-2

**Published:** 2023-03-28

**Authors:** Isabel Kreis, Lei Zhang, Matthias Mittner, Leonard Syla, Claus Lamm, Gerit Pfuhl

**Affiliations:** 1 Department of Psychology, UiT – The Arctic University of Norway, Tromsø, Norway; 2 NORMENT, Institute of Clinical Medicine, University of Oslo, Oslo, Norway; 3 Institute for Systems Neuroscience, University Medical Center Hamburg-Eppendorf, Hamburg, Germany; 4 Centre for Human Brain Health, School of Psychology, University of Birmingham, Birmingham, UK; 5 Institute for Mental Health, School of Psychology, University of Birmingham, Birmingham, UK; 6 Social, Cognitive and Affective Neuroscience Unit, Department of Cognition, Emotion, and Methods in Psychology, Faculty of Psychology, University of Vienna, Vienna, Austria; 7 Vienna Cognitive Science Hub, University of Vienna, Vienna, Austria; 8 Department of Psychology, Norwegian University of Science and Technology, Trondheim, Norway

**Keywords:** Volatility, Belief updating, Uncertainty, Autism, Psychosis, Pupillometry, Hidden Markov model

## Abstract

Aberrant belief updating due to misestimation of uncertainty and an increased perception of the world as volatile (i.e., unstable) has been found in autism and psychotic disorders. Pupil dilation tracks events that warrant belief updating, likely reflecting the adjustment of neural gain. However, whether subclinical autistic or psychotic symptoms affect this adjustment and how they relate to learning in volatile environments remains to be unraveled. We investigated the relationship between behavioral and pupillometric markers of subjective volatility (i.e., experience of the world as unstable), autistic traits, and psychotic-like experiences in 52 neurotypical adults with a probabilistic reversal learning task. Computational modeling revealed that participants with higher psychotic-like experience scores overestimated volatility in low-volatile task periods. This was not the case for participants scoring high on autistic-like traits, who instead showed a diminished adaptation of choice-switching behavior in response to risk. Pupillometric data indicated that individuals with higher autistic- or psychotic-like trait and experience scores differentiated less between events that warrant belief updating and those that do not when volatility was high. These findings are in line with misestimation of uncertainty accounts of psychosis and autism spectrum disorders and indicate that aberrancies are already present at the subclinical level.

**Supplementary Information:**

The online version contains supplementary material available at 10.3758/s13415-023-01088-2.

Making good decisions requires learning about the probabilistic risks^1^ associated with different choices, i.e., learning which choices are most likely associated with a positive outcome, and updating beliefs about these risks if they change. To illustrate, imagine Lisa, who loves apples. There are two grocery stores in Lisa’s neighborhood, store A and store B, and over time Lisa has learned that the probability to obtain good apples, i.e., the risk, is approximately 80% at store A but only 20% at store B. Hence, she will continue shopping at store A. Unbeknownst to Lisa, one day the owners of both stores swap suppliers, resulting in better apples now being more likely at store B than store A. Lisa will have to learn about this change in risk through experience and adapt her behavior accordingly if she wants to continue making good decisions. The ability to make good decisions while taking the statistical properties of the environment, including potential changes, into account can be impaired in psychiatric disorders. Accordingly, maladaptive and increased choice-switching during reversal learning tasks (where the risk associated with the choice options reverses over time) has been observed in both autism spectrum disorders (D'Cruz et al., 2013; Mussey et al., 2015; Solomon et al., 2011) and psychotic disorders, such as schizophrenia (Culbreth et al., 2016; Kaplan et al., 2016; Li et al., 2014; Murray et al., 2008; Schlagenhauf et al., 2014; Waltz et al., 2013). In these tasks, similar to the example of Lisa, participants are presented with different choice options that are associated with a positive outcome with a specific probability or risk, but this risk changes over time. This introduces several levels of uncertainty to the task environment. First, there is the irreducible risk of a choice-outcome association (e.g., an 80:20 chance of a positive outcome following choice of option A). Second, because this risk is unknown and has to be learned through experience (by making choices and observing the outcome), there is estimation uncertainty, i.e., uncertainty about the accuracy of one’s own risk estimation. Estimation uncertainty is highest in the beginning of a new learning sequence and can additionally be increased by volatility of the learning environment, which is the rate at which risk changes (e.g., risk might change from 80:20 to 20:80 every 12 ± 5 task trials). If volatility is high, risk changes often and unpredictably; if it is low, such changes happen more rarely.

The findings of maladaptive and increased choice-switching during reversal learning tasks in autism and schizophrenia may be explained by an overestimation of this volatility (Cole et al., 2020; Deserno et al., 2020; Lawson et al., 2017). Evidently, an elevated belief about volatility may increase a person’s tendency to switch between two choices, as the probability for obtaining a reward for one choice over another might change over time. Consequentially, beliefs about risk are updated at a higher rate, and behavior becomes hyperflexible (Deserno et al., 2020). However, some findings imply that individuals with autism do not overestimate volatility per se but are more sensitive to it, with impaired performance under volatile as opposed to stable conditions (Goris et al., 2020; Robic et al., 2015). In addition, choice behavior is affected by the accuracy with which risk is learned and represented in the first place, which seems to be diminished in patients with schizophrenia (Murray et al., 2008; Waltz et al., 2013; Weickert et al., 2010) and individuals with autism (Solomon et al., 2015). Taken together, this indicates aberrancies in the representation and processing of different types of uncertainty in both clinical groups. Interestingly, misestimation of uncertainty and skewed belief updating have been proposed to play a major role in the development of symptoms in both autism and psychotic disorders within the Bayesian brain framework (Fletcher & Frith, 2009; Van de Cruys et al., 2014; van Schalkwyk et al., 2017). Specifically, symptoms may arise from false inferences about the world, which in turn are caused by alterations in ascribing uncertainty to prior beliefs and new sensory information (Adams et al., 2013; Powers et al., 2017). This may lead to delusions and hallucinations in psychosis (Adams et al., 2013; Fletcher & Frith, 2009) and may cause symptoms of sensory overload and oversensitivity to sensory stimulation in autism (Lawson et al., 2014).

Subjective representations of the different uncertainties that characterize a task environment can be captured with cognitive-computational models fitted to observed behavior, e.g., Bayesian inference models, such as the Hidden Markov Model (HMM), which has successfully been applied to reversal learning tasks (Hämmerer et al., 2019; Schlagenhauf et al., 2014). In the HMM, subjective volatility is reflected by the transition probability, which describes the probability to switch between different state beliefs about risk within the task. On any given task trial, a state belief reflects the belief of being in a certain state of the task, e.g., one where option B is more beneficial than option A with an associated risk of 80:20. Based on those state beliefs, two task-relevant trial-wise latent variables can be derived: choice uncertainty and Bayesian surprise. Choice uncertainty reflects the degree of uncertainty surrounding the belief that the choice made on a given trial will lead to a positive outcome. It is high under high risk, i.e., when the probability of a positive outcome following a particular choice is similar to the probability of a negative outcome, and when estimation uncertainty of the current risk is high (e.g., at the beginning of a new learning sequence or under high volatility). Bayesian surprise expresses the extent to which a current state belief should be updated in the face of new evidence, i.e., a new choice-outcome observation, and is particularly high under high volatility (Hämmerer et al., 2019) where rapid changes in risk can result in very unexpected outcomes following previously beneficial choices. Both uncertainty and surprise can prompt belief updating, indicating a general need to “learn more” (to reduce uncertainty) and “how much more” (depending on the size of the surprise), respectively.

Neurobiologically, uncertainty is thought to be encoded by neuromodulatory systems, where contextual change resulting from volatility may specifically be signaled by norepinephrine (Friston et al., 2006; Yu & Dayan, 2005). This fits well with accounts linking norepinephrinergic activity in the locus coeruleus (the LC-NE system) to explorative behavior (Aston-Jones & Cohen, 2005), given that contextual change promotes belief updating through exploration and learning. Activity in the LC-NE system can be indexed through pupil size (Joshi et al., 2016; Rajkowski et al., 1994; Samuels & Szabadi, 2008), which in turn has been found to respond to (choice) uncertainty (Kreis et al., 2021; Nassar et al., 2012), volatility (Browning et al., 2015; Lawson et al., 2017), surprise, and belief updating (Hämmerer et al., 2019; Preuschoff et al., 2011). This response possibly reflects an upregulation of neural gain and learning (Eldar et al., 2013) to reduce uncertainty about current task states and update beliefs accordingly. Pupil responses in individuals with autism (Lawson et al., 2017) or schizophrenia (Kreis et al., 2021; Steinhauer et al., 1979; Steinhauer & Zubin, 1982), however, seem to scale less with uncertainty or surprise, suggesting a reduced ability to differentiate events that warrant a belief update from those that do not and to regulate neural gain accordingly. Notably, the extent to which pupil size scales with learning signals, such as choice uncertainty and Bayesian surprise, may depend on the task environment. In a volatile task environment, high choice uncertainty may be attributed primarily to estimation uncertainty, i.e., limited knowledge about the current risk associated with the different choice options. Hence, more attention may be devoted to the presented outcomes and pupil size may track choice uncertainty more closely. In a stable task environment, high choice uncertainty may be attributed primarily to risk. Because this risk, once learned, is irreducible, new outcomes may be less informative and pupil size may scale less with choice uncertainty. The same is true for Bayesian surprise, which may indicate a change in risk under volatile conditions but might simply reflect task-inherent statistical deviations under stable conditions.

The diminished pupil responses to uncertainty and surprise that have been observed in autism and schizophrenia may be caused by a failure to represent such task structures appropriately (Hämmerer et al., 2019), e.g., by misestimating risk, enhanced estimation uncertainty due to diminished learning, or overestimation of volatility, which renders all new events similarly surprising and relevant for belief updating and learning. It is unclear to what extent these findings translate to neurotypical populations varying naturally on autistic- and psychotic-like symptoms (Abu-Akel et al., 2015; Yung et al., 2009) and whether the effects scale with symptom load. The study of subclinical populations is essential when evaluating the potential role of uncertainty misestimation for symptom development as described above. Furthermore, findings in patient samples may be tainted by the effects of anticholinergic medication on pupil size (Naicker et al., 2016). Hence, the present study tested whether autistic- and psychotic-like traits and experiences assessed in a neurotypical sample are associated with similarly reduced pupil responses to events that should promote belief updating. Such an association may indicate an increased exploratory processing style, possibly resulting from elevated subjective volatility. Using a probabilistic prediction task with different volatility and risk conditions, latent computational variables (subjective volatility, choice uncertainty, Bayesian surprise) were derived from computational models and tested for their relationship with trait and experience scores and with changes in pupil size. Trait and experience scores as well as task conditions were further investigated in their relation to observable behavior, such as accuracy of and switching between predictions, both of which were expected to differ depending on the degrees of volatility and risk. Interaction effects between these task-related uncertainty conditions and trait scores would then help to clarify whether different traits are related to issues in dealing with volatility (e.g., reflected in particularly decreased performance in the volatile condition), misestimation of volatility (e.g., reflected in similar amounts of switching under more and less volatile conditions), or misestimation of risk (e.g., reflected in similar amounts of switching under high and low risk conditions). Working memory capacity was evaluated to ensure that trait-related differences in probabilistic learning were not driven by differences in executive functioning and working memory resources, which have been linked to learning about probabilities and task structures (Deserno et al., 2020; Waltz & Gold, 2007).

## Method

### Participants

Participants were recruited through pamphlets, social media, and from university classes. Inclusion criteria were: (1) 18–60 years of age; (2) normal or corrected-to-normal eyesight; (3) no history of neurological disorders; (4) no acute psychiatric disorder, (5) no substance dependence, and (6) no intake of any psychoactive medication or recreational drug within 3 months prior to the assessment. The final sample consisted of 52 individuals and is described in Table 1. The sample size was based on a power analysis (α = 0.05, two-sided, 1-β = 0.8) leaned on recent findings of an association between pupil response to uncertainty and performance in a volatile task environment (r = 0.62, N = 22; de Berker et al., 2016). Effect size and final sample size were slightly reduced (to 0.4) and increased (to N = 52) respectively to account for publication bias and take potential participant exclusion due to eye-tracking data quality into account. For all participants, written, informed consent was obtained prior to the assessment. The study was conducted in accordance with the guidelines of the Declaration of Helsinki and approved by the internal ethics committee of the Department of Psychology at UiT – The Arctic University of Norway (reference number: 2017/1912).

**Table 1:** Summary statistics of demographic variables, average questionnaire scores, and working memory capacity

	*n*	*M (SD)*	*Md (IQR)*
Gender (f/m)	31/21		
Education (HS/BA/MA)	34/13/5		
Age		23.50 (4.13)	22.50 (6.00)
CAPE-P		1.46 (0.30)	1.41 (0.50)
AQ		0.30 (0.15)	0.29 (0.21)
WMC			
	Verbal-numerical	3.92 (0.90)	4.00 (1.00)
	Visual-spatial	6.13 (0.84)	6.00 (1.25)

*n* = sample size of the different levels of categorical variables; *M* = mean; *SD* = standard deviation; *Md* = median; *IQR* = interquartile range; f = female, m = male; HS = high school, BA = bachelor, MA = master; CAPE-P = average score of the positive dimension scale of the CAPE (Community Assessment of Psychic Experiences), maximum score = 4, minimum = 1; AQ = average score of the Autism Quotient, maximum score = 1, minimum = 0; WMC = working memory capacity score, maximum score = 7, minimum = 0. Results are rounded to two decimal places

### Materials and procedure

#### Probabilistic prediction task

**Fig. 1:**
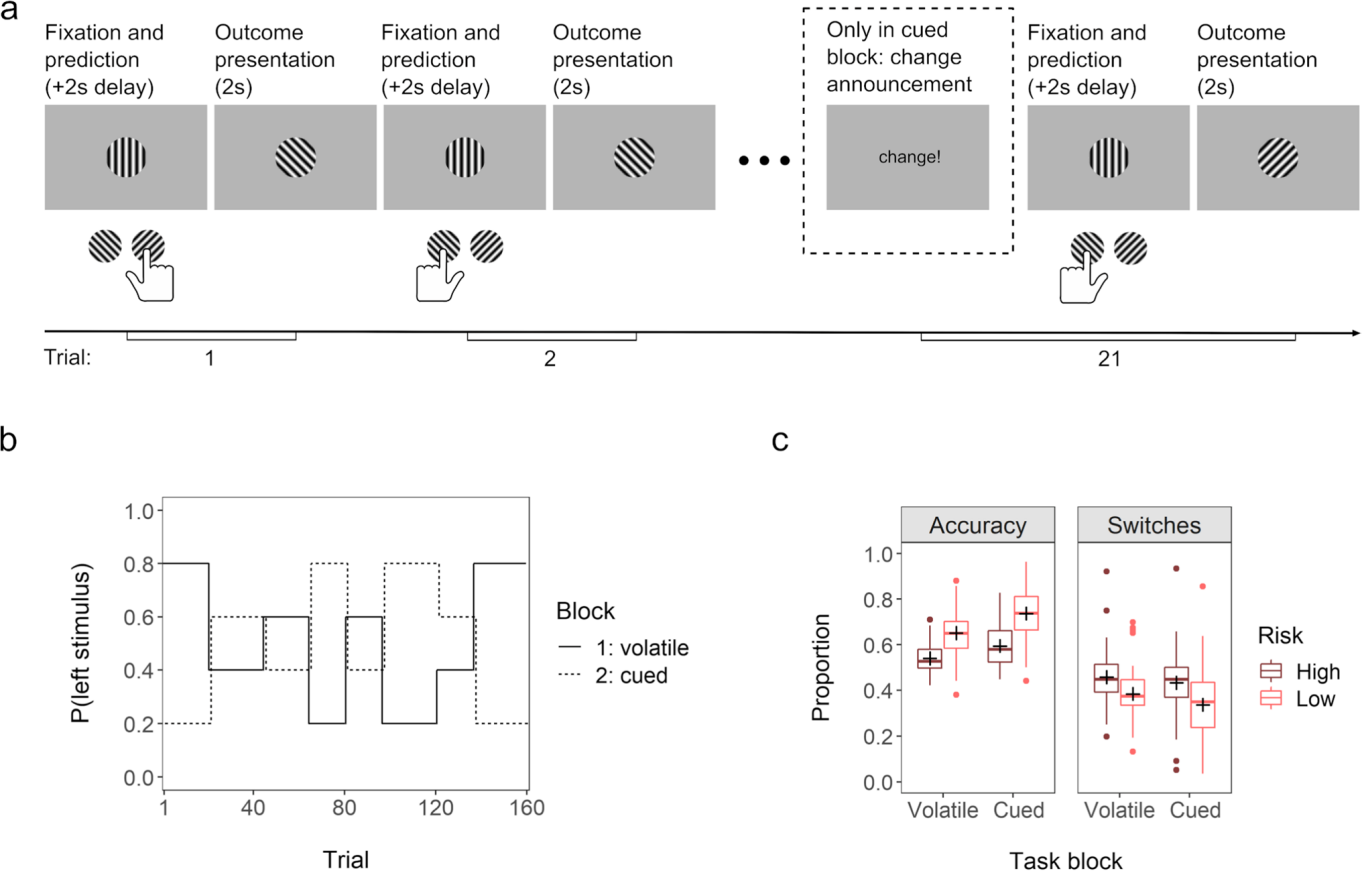
Probabilistic prediction task. *Notes*. Figure adapted from Kreis et al. (2021). (**a**) Example trials with a change of stimulus probabilities on trial 21. In the second, cued task block, this change was preceded by a “change” message on screen. In response, participants had to press “enter” before they could continue with the task. (**b**) Task structure: probabilities for the left- (p_left_) and the right-tilted (1-p_left_) stimulus in the first task block (*volatile block*; solid line) and the second task block (*cued block*; dashed line). Time points of changes were identical in both blocks (lines are slightly jittered for better readability), as was the order of the different risk conditions. The identity of the majority stimulus in the different risk conditions was inverted in the second as opposed to the first task block. (**c**) Boxplots displaying the proportion of trials where the majority stimulus was predicted (accuracy) and where choices differed from those on the preceding trial (switches) for the different task blocks and risk conditions, respectively. Means are displayed as crosses

A probabilistic prediction task programmed in Psychopy (Peirce et al., 2019) was administered to measure decision-making and belief updating under different uncertainty conditions. Two task blocks (“volatile” and “cued”) differed in their degree of volatility, compromising 160 trials each (+ 12 and 18 training trials for the volatile and the cued task block, respectively). On each trial, a vertically striped stimulus was presented in the center of the screen, followed by an either left- or right-tilted stimulus (orientation ± 45°). Upon presentation of the vertical stimulus, participants had to indicate via keypress (left-alt: “left-tilted”, right-ctrl: “right-tilted”) which one of the two tilted stimuli they predicted to see next (Fig. 1a). After a 2-second delay, the outcome (left- or right-tilted stimulus) was displayed for 2 seconds. The probability of seeing either a left- or a right-tilted stimulus was unknown to the participants and changed every 20 (±4) trials, alternating between 80:20 and 60:40 and their reverse (20:80, 40:60), providing task periods of high (60:40, 40:60) and low (80:20, 20:80) risk and inducing high estimation uncertainty after each change (Fig. 1b). Changes in the underlying distribution of left- and right-tilted stimuli were not announced in the volatile and announced in the cued block (Fig. 1a). The order of the different risk conditions was the same across blocks and participants, while the identity of the majority stimulus was inverted between blocks (Fig. 1b). Block order was not counterbalanced, with the volatile task block always administered first. This was done to prevent priming participants from detecting the hidden change points in the volatile task block and maximize the experience of this block as volatile. If the cued task block were to be presented first, participants might form expectations about the timing of the hidden change points in the subsequent volatile block. Because the timing of these change points was identical across blocks, this would reduce unpredictability in the volatile block and might diminish the subjective experience of volatility overall.

Participants were instructed to fixate the center of the screen throughout the task and minimize the total amount of prediction errors. They were informed that over several trials in a row either the left- or the right-tilted stimulus would appear more often with a fixed but unknown probability, that probability and identity of the majority stimulus might change repeatedly, and that these changes would be hidden in the first, but announced in the second task block. Participants were advised to forget all they had learned about the stimulus probabilities on previous trials and start to learn “anew” following a change announcement. Task performance was assessed as accuracy (relative frequency of predicting the current majority stimulus) and proportion of choice switches (proportion of times where prediction on trial *t* + 1 was different from prediction on trial *t*; Fig. 1c), both aggregated separately for the two risk conditions per block.

#### Questionnaires and working memory measure

Autistic-like traits and psychotic-like experiences were measured with the abridged version of the Autism Quotient (AQ; Hoekstra et al., 2011), and the positive symptom dimension of the Community Assessment of Psychic Experiences (CAPE-P; Stefanis et al., 2002), respectively. For four participants, responses to three items of the CAPE-P (15 % of all items) were missing; thus, CAPE-P and AQ average scores are used in the main analyses. Additionally, age, gender, and education (recorded in categories of highest completed degree: high school, Bachelor, Master) were recorded. Working-memory capacity was assessed in the verbal-numerical domain and the visual-spatial domain, using the digit span and the matrix span task of a computerized open source working memory test battery (Stone & Towse, 2015; see Supplementary Methods for details).

#### Pupil size

During the prediction task, pupil diameter was recorded from the right eye at a sampling rate of 500 Hz with an infrared video-based eye tracker (Eyelink 1000, SR Research).

#### Procedure

On the day of the assessment, participants signed the consent form before completing the first block of the prediction task (ca. 15 min). Then, the working memory task (ca. 10 min), a decision-making task (ca. 5 min; results are not reported here and are not assumed to affect behavior on any of the other tasks), and the second block of the prediction task (ca. 15 min) were administered. Finally, participants completed the questionnaires (ca. 10 min).

### Analysis

#### Computational modelling of behavior

To quantify latent cognitive processes, seven candidate computational models were fitted independently to participants’ choices for the volatile and the cued block of the prediction task, respectively. The models included a simple win-stay-lose-shift model (Worthy & Todd Maddox, 2014), four different Reinforcement Learning models (den Ouden et al., 2013; Gläscher et al., 2008; Pearce & Hall, 1980; Rescorla & Wagner, 1972) and two variants of the Hidden Markov Model (HMM; Schlagenhauf et al., 2014). All models were estimated under the hierarchical Bayesian framework (Ahn et al., 2017; Gelman et al., 2013; Zhang et al., 2020) using a Hamiltonian Monte Carlo (HMC) method within the statistical language Stan. See Supplementary Methods for details, including model comparison and rationale behind the choice of models. Model comparison revealed that a variant of the HMM provided the best fit for both task blocks (see Supplementary Tables S1 and S2). The HMM is a Bayesian inference model, which assumes that participants make their choices (i.e., predict “left” or “right”) based on their belief of being in a state of the task where either the left- or the right-tilted stimulus is more common. Those beliefs are updated on a trial-by-trial basis, modulated by the history of action-outcome pairs and the estimated transition probability for the two states (i.e., how likely does the state change from “predominantly left” to “predominantly right-tilted stimuli” and vice versa). Crucially, the transition probability γ reflects the perceived, i.e., subjective, volatility of the task environment. In the winning variant of the HMM, effects of positive (prediction correct) versus negative feedback (prediction incorrect) on state belief updates were allowed to differ (HMM_RP_; Schlagenhauf et al., 2014). For the cued task block, this model included belief resets at every announced change point.

Based on the HMM_RP_’s trial-wise state beliefs both before $\left(P\left(S_{t_{pre}}\right)\right)$ and after observing the outcome $\left(P\left(S_{t_{post}}\right)\right)$, a Bayesian surprise signal was estimated as their Kullblack-Leibler (KL) divergence:

1 $${D}_{KL}\left(P\left({S}_{t_{post}}\right)\Big\Vert P\left({S}_{t_{pre}}\right)\right)=\sum_{i=1}^2P\left({S}_{t_{pre}}=i\right)\log \left(\frac{P\left({S}_{t_{post}}=i\right)}{P\left({S}_{t_{pre}}=i\right)\ }\right)$$

This expresses the extent to which the internal model (i.e., belief about the state) should be updated on each trial, with *S*_*t*_ = *i* denoting state *i* of the two different states (state “predominantly left” and state “predominantly right-tilted stimuli”).

Similarly, choice uncertainty regarding the chosen stimulus was derived for different posterior “reward” (i.e., a correct prediction) probabilities as belief entropy:

2 $$H\left({S}_t\right)=-\sum_{i=1}^2P\left({S}_t=i\right)\log P\left({S}_t=i\right)$$

#### Pupil signal preprocessing

Eye blinks and other artifacts (e.g., caused by head movements or eye lid flickering) were detected with a custom-built filter based on the pupil signal’s velocity implemented in R (version 3.5.1; R Core Team, 2018) and were removed through cubic-spline interpolation (Mathôt et al., 2018). Velocity thresholds and margins for blink windows were adapted on an individual basis to account for inter-individual differences in blink characteristics, e.g., regarding the speed of signal recovery. The corrected pupil signal was smoothed with a low pass Butterworth filter using a cut-off frequency of 3 Hz, because high-frequency components are more likely caused by noise (Klingner et al., 2008). When the time window of interpolation spanned more than 1,000 consecutive milliseconds, the signal was treated as missing. The smoothed pupil signal was z-scored per block and participant and baseline-corrected per trial by subtracting the average signal of the 500 ms preceding the outcome onset. Trials with more than 50% of interpolated and missing data within the baseline or outcome presentation time window were treated as missing in subsequent analyses, where maximum pupil dilation during outcome presentation was the main variable of interest.

#### Statistical analyses

Linear mixed-effects models were used to investigate the effect of task conditions (high risk: 60:40/40:60 trials vs. low risk: 80:20/20:80 trials; cued block vs. volatile block), AQ and CAPE-P scores on accuracy, choice switches, choice uncertainty and Bayesian surprise. In all models, nested random factors were specified, allowing for different intercepts at the different levels of risk condition within blocks nested within participants. The effect of choice uncertainty, Bayesian surprise, AQ, and CAPE-P scores on pupil dilation also were tested by using linear mixed-effects models. Model residuals were tested for normality and dependent variables were cube-root (Bayesian surprise) or square-root transformed (maximum pupil dilation) when this assumption was violated. Because autistic traits and psychotic-like experiences are positively correlated (Bevan Jones et al., 2012; Martinez et al., 2020), analyses were implemented separately for AQ and CAPE-P. Nonnormally distributed variables were identified with Shapiro-Wilk tests and to evaluate the relationship between questionnaire scores and potential covariates, Spearman correlations (age; verbal-numerical and visual-spatial working memory scores), Kruskal-Wallis (education) and Mann-Whitney *U* tests (gender) were performed. Data were analyzed with the statistical programming language R (version 3.5.1; R Core Team, 2018), with R package *nlme* (version 3.1-152; Pinheiro et al., 2021) for linear-mixed effects models and *ggplot2* (version 3.3.5, Wickham, 2016) for visualization. All testing was conducted two-sided and with a significance level of 0.05. Standardized regression coefficients are reported together with 95% confidence intervals. All results are rounded to two decimal places.

## Results

AQ and CAPE-P scores were positively but not significantly correlated (ρ = 0.25, *p* = 0.08). Neither demographic nor working-memory variables were related to questionnaire scores (see Supplementary Results) and therefore were not included as covariates in any of the subsequent analyses.

### Accuracy differs by task conditions but not questionnaire scores

Accuracy was higher in the cued task block (β = 0.69, *t* = 5.40, *p* < 0.001, 95% CI [0.43, 0.94]) and lower in the high-risk condition (β = −0.90, *t* = −8.57, *p* < 0.001, [−1.10, −0.69]; Fig. 1c), with no significant interaction between block and risk (β = −0.25, *t* = −1.69, *p* = 0.09, [−0.54, 0.04]). When included in the model, none of the AQ predictors yielded a significant effect (AQ: β = −0.10, *t* = −0.92, *p* = 0.36, [−0.33, 0.12]; block*AQ: β = 0.04, *t* = 0.29, *p* = 0.77, [−0.22, 0.29]; risk*AQ: β = 0.08, *t* = 0.78, *p* = 0.44, [−0.12, 0.29]; block*risk*AQ: β = −0.16, *t* = −1.06, *p* = 0.29, [−0.45, 0.13]; Figure S1). Results of a model that included CAPE-P instead of AQ scores were similar (CAPE-P: β = −0.12, *t* = −1.06, *p* = 0.29, [−0.34, 0.10]; block*CAPE-P: β = 0.00, *t* = −0.01, *p* = 0.99, [−0.25, 0.25]; risk*CAPE-P: β = 0.17, *t* = 1.68, *p* = 0.10, [−0.03, 0.38]; block*risk*CAPE-P (β = −0.23, *t* = −1.59, *p* = 0.12, [−0.52, 0.05]; Figure S1).

### Choice switches differ by task conditions and AQ scores

**Fig. 2:**
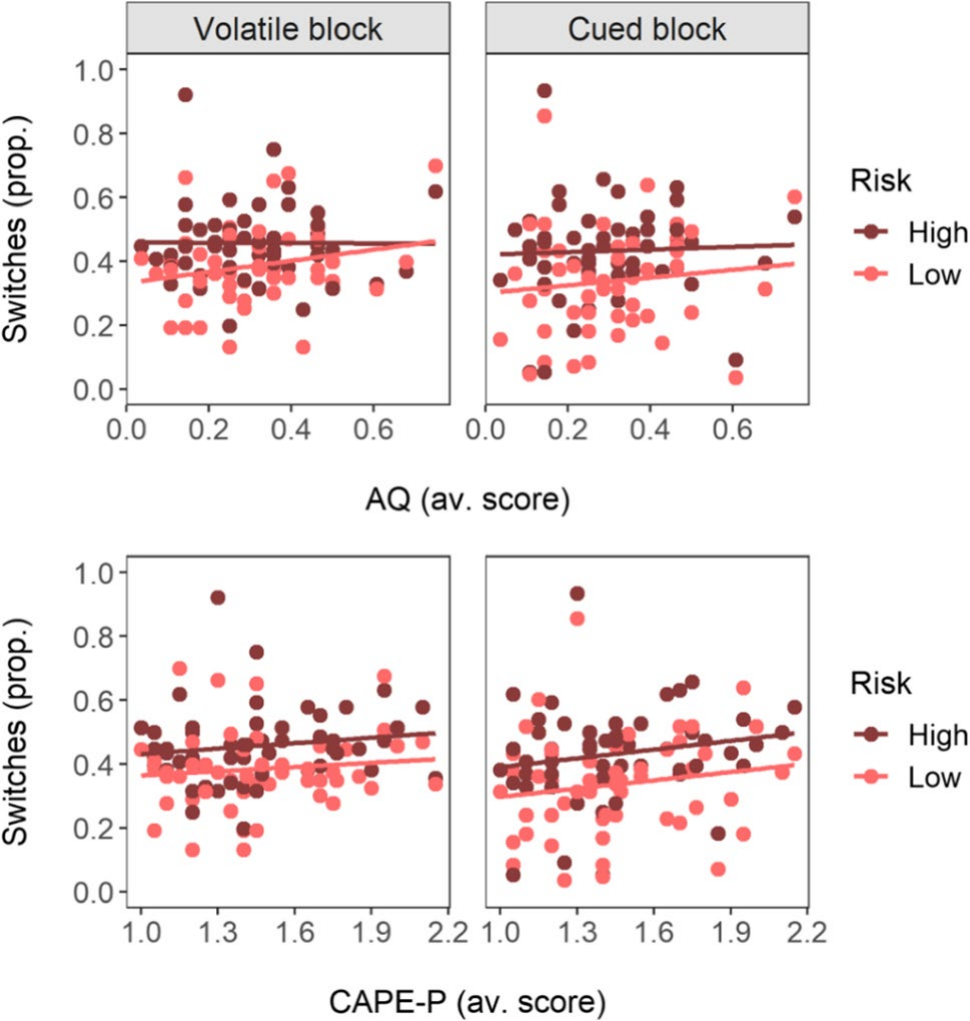
Relationship between trait and experience scores and proportion of choice switches. *Notes*. Proportion of choice switches is presented separately for the different task blocks (columns) and risk conditions (color). Trait and experience scores are average scores of AQ (top row) and CAPE-P (bottom row). Points represent values per participant and task condition; lines are regression lines (linear model) to demonstrate trends. Proportion of choice switches was higher under high risk than low risk conditions. This association was moderated by AQ scores, with decreasing differentiation between high and low risk trials as AQ scores increased (see top two panels; interaction effect risk*AQ: β = −0.19, *p* = 0.03)

Proportion of choice switches was lower in the cued task block (β = −0.33, *t* = −2.76, *p* < 0.01, 95% CI [−0.56, −0.09]) and higher for high-risk trials (β = 0.50, *t* = 5.80, *p* < 0.001, [0.33, 0.67]; Fig. 1c), with no significant interaction between block and risk (β = 0.16, *t* = 1.27, *p* = 0.21, [−0.08, 0.40]). Inclusion of AQ scores revealed a significant interaction with risk (β = −0.19, *t* = −2.22, *p* = 0.03, [−0.36, −0.02]), indicating that proportion of switches on high- versus low-risk trials differed less for participants scoring higher on the AQ (Fig. 2). Other AQ predictors were not significant (AQ: β = 0.18, *t* = 1.39, *p* = 0.17, [−0.08, 0.45]; block*AQ: β = −0.06, *t* = −0.47, *p* = 0.64, [−0.29, 0.18]; block*risk*AQ: β = 0.11, *t* = 0.87, *p* = 0.39, [−0.13, 0.34]). None of the CAPE-P predictors were significant (CAPE-P: β = 0.09, *t* = 0.69, *p* = 0.49, [−0.17, 0.35]; block*CAPE-P: β = 0.09, *t* = 0.73, *p* = 0.47, [−0.15, 0.32]; risk*CAPE-P: β = 0.03, *t* = 0.30, *p* = 0.77, [−0.14, 0.20]; block*risk*CAPE-P: β = −0.02, *t* = −0.13, *p* = 0.90, [−0.26, 0.23]; Fig. 2).

### Transition probability (HMM_RP_) differs by block and CAPE-P scores

**Fig. 3:**
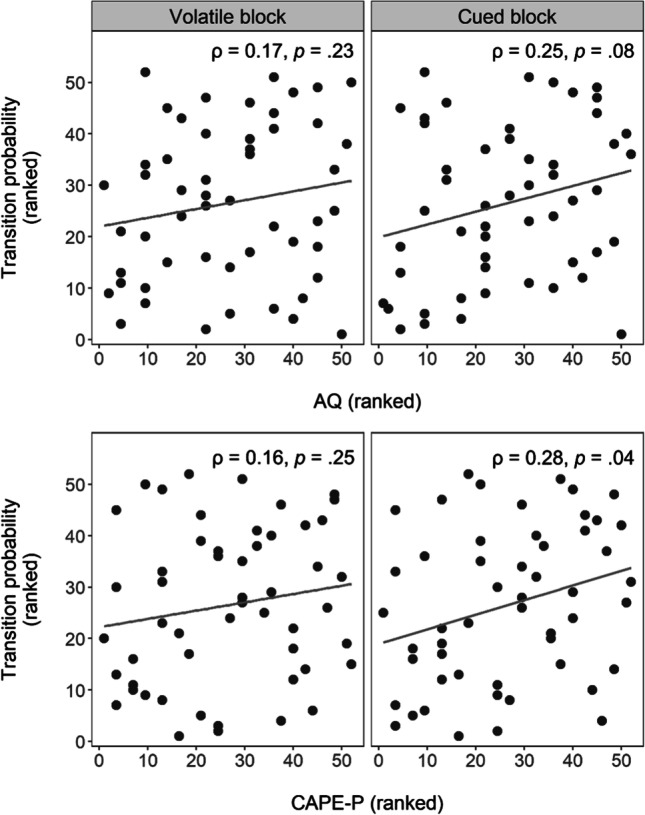
Relationship between trait and experience scores and subjective volatility (transition probability). *Notes*. Subjective volatility estimates (transition probability) are plotted against trait and experience scores of AQ (top row) and CAPE-P (bottom row), separately for the different task blocks (columns). In accordance with the non-normal distributions of those variables, Spearman correlations are used and ranked values are presented. Statistics of the Spearman correlations are displayed in the top-right corner of each panel, and regression lines (linear model) are added to demonstrate trends

Because HMM_RP_ models were fitted separately per task block and differed slightly in terms of belief resets, the estimated transition probabilities were contrasted by directly comparing their posterior distributions. Transition probability γ (subjective volatility) was credibly higher in the volatile block (*M* = 0.22) than in the cued block (*M* = 0.10) of the task (95% highest density interval of the difference [0.05, 0.19]). AQ scores were not significantly related to γ in either block (volatile block: ρ = 0.17, *p* = 0.23; cued block: ρ = 0.25, *p* = 0.08), whereas CAPE-P scores and γ correlated positively within the cued block (ρ = 0.28, *p* = 0.04; volatile block: ρ = 0.16, *p* = 0.25), indicating that participants with more psychotic-like experiences assumed higher volatility in the low-volatile block (Fig. 3).

### Pupil response to choice uncertainty and Bayesian surprise is modulated by AQ and CAPE-P scores

Both choice uncertainty (entropy) and Bayesian surprise differed by task conditions but were unrelated to questionnaire scores. Both measures had higher values under high risk, Bayesian surprise was increased under high volatility, and the effect of risk on choice uncertainty was more pronounced under low volatility (see Supplementary Results). The pupil response to trial-by-trial choice uncertainty and Bayesian surprise (z-scored per participant and block) was assessed in two separate models. Here, maximum pupil dilation during outcome presentation (square root transformed) was significantly larger on trials where choice uncertainty was higher (β = 0.02, *t* = 2.16, *p* = 0.03, 95% CI [0.00, 0.05]), independent of block (block: β = 0.06, *t* = 1.26, *p* = 0.22, [−0.04, 0.17]; choice uncertainty*block: β *=* 0.01, *t* = 0.41, *p* = 0.68, [−0.03, 0.04]), and similarly, increased with Bayesian surprise (β = 0.04, *t* = 3.22, *p* < 0.01, [0.01, 0.06]), with no block effects (block: β *=* 0.06, *t* = 1.25, *p* = 0.22, [−0.04, 0.17]; Bayesian surprise*block: β = −0.03, *t* = −1.55, *p* = 0.12, [−0.06, 0.01]). However, these effects changed when adding questionnaire scores to the models, with responses to Bayesian surprise but not choice uncertainty affected by AQ (Table 2) and responses to choice uncertainty but not Bayesian surprise affected by CAPE-P (Table 3).

**Table 2:** Linear mixed-effects model results for pupil dilation (dependent variable) by choice uncertainty (entropy), Bayesian surprise and AQ

Latent HMM_RP_ predictor		β	*t*	*p*	95% CI	*R*^*2*^_*M*_	*R*^*2*^_*C*_
*Entropy*						0.01	0.16
	Entropy	0.02	2.16	0.03	[0.00, 0.05]		
	Block	0.06	1.30	0.20	[−0.03, 0.16]		
	AQ	−0.01	−0.14	0.89	[−0.12, 0.11]		
	Entropy*block	0.01	0.41	0.68	[−0.03, 0.04]		
	Entropy*AQ	0.00	−0.06	0.95	[−0.02, 0.02]		
	Block*AQ	0.10	1.99	0.05	[0.00, 0.19]		
	Entropy*block*AQ	0.01	0.47	0.64	[−0.02, 0.04]		
*Bayesian surprise*						0.01	0.16
	Bay. surprise	0.04	3.24	<0.01	[0.01, 0.06]		
	Block	0.06	1.30	0.20	[−0.03, 0.16]		
	AQ	−0.01	−0.14	0.89	[−0.12, 0.11]		
	Bay. surprise*block	−0.03	−1.57	0.12	[−0.06, 0.01]		
	Bay. surprise*AQ	−0.02	−2.17	0.03	[−0.05, 0.00]		
	Block*AQ	0.10	1.98	0.05	[0.00, 0.19]		
	Bay. surprise*block*AQ	0.05	3.07	<0.01	[0.02, 0.08]		

Coefficients of the fixed effects in the separate linear mixed-effects models of pupil dilation by entropy and Bayesian surprise (both z-scored per block and participant; model included nested random effects for block within participant), including AQ score as a predictor. CI = confidence interval; block = contrast of the second, cued task block to the first, volatile task block; AQ = Autism Quotient average score; *R*^*2*^*m* = marginal *R*^*2*^, i.e*.* proportion of variance explained by the fixed effects alone; *R*^*2*^*c* = conditional *R*^*2*^, i.e., proportion of variance explained by both the fixed and random effects (*R*^*2*^*m* and *R*^*2*^*c* based on Nakagawa & Schielzeth, 2013). Results are rounded to two decimal places

**Table 3:** Linear mixed-effects model results for pupil dilation by choice uncertainty (entropy), Bayesian surprise and CAPE-P (C-P)

Latent HMM_RP_ predictor		β	*t*	*p*	95% CI	*R*^*2*^_*M*_	*R*^*2*^_*C*_
*Entropy*						0.01	0.16
	Entropy	0.02	2.05	0.04	[0.00, 0.05]		
	Block	0.06	1.26	0.21	[−0.04, 0.17]		
	C-P	−0.11	−2.07	0.04	[−0.23, 0.00]		
	Entropy*block	0.01	0.50	0.62	[−0.02, 0.04]		
	Entropy*C-P	-0.02	−1.77	0.08	[−0.04, 0.00]		
	Block*C-P	0.01	0.21	0.83	[−0.09, 0.11]		
	Entropy*block*C-P	0.04	2.22	0.03	[0.00, 0.07]		
*Bayesian surprise*						0.01	0.16
	Bay. surprise	0.04	3.21	<0.01	[0.01, 0.06]		
	Block	0.06	1.25	0.22	[−0.04, 0.17]		
	C-P	−0.11	−2.07	0.04	[−0.23, 0.00]		
	Bay. surprise*block	−0.03	−1.53	0.13	[−0.06, 0.01]		
	Bay. surprise*C-P	0.00	−0.17	0.86	[−0.03, 0.02]		
	Block*C-P	0.01	0.21	0.84	[−0.09, 0.11]		
	Bay. surprise*block*C-P	0.02	1.40	0.16	[−0.01, 0.06]		

Coefficients of the fixed effects in the separate linear mixed-effects models of pupil dilation by entropy and Bayesian surprise (both z-scored per block and participant; model included nested random effects for block within participant), including C-P score as a predictor. CI = confidence interval; block = contrast of the second, cued task block to the first, volatile task block; C-P = average score of the positive dimension scale of the CAPE (Community Assessment of Psychic Experiences); *R*^*2*^*m* = marginal *R*^*2*^, i.e*.* proportion of variance explained by the fixed effects alone; *R*^*2*^*c* = conditional *R*^*2*^, i.e. proportion of variance explained by both the fixed and random effects (*R*^*2*^*m* and *R*^*2*^*c* based on Nakagawa & Schielzeth, 2013). Results are rounded to two decimal places

**Fig 4:**
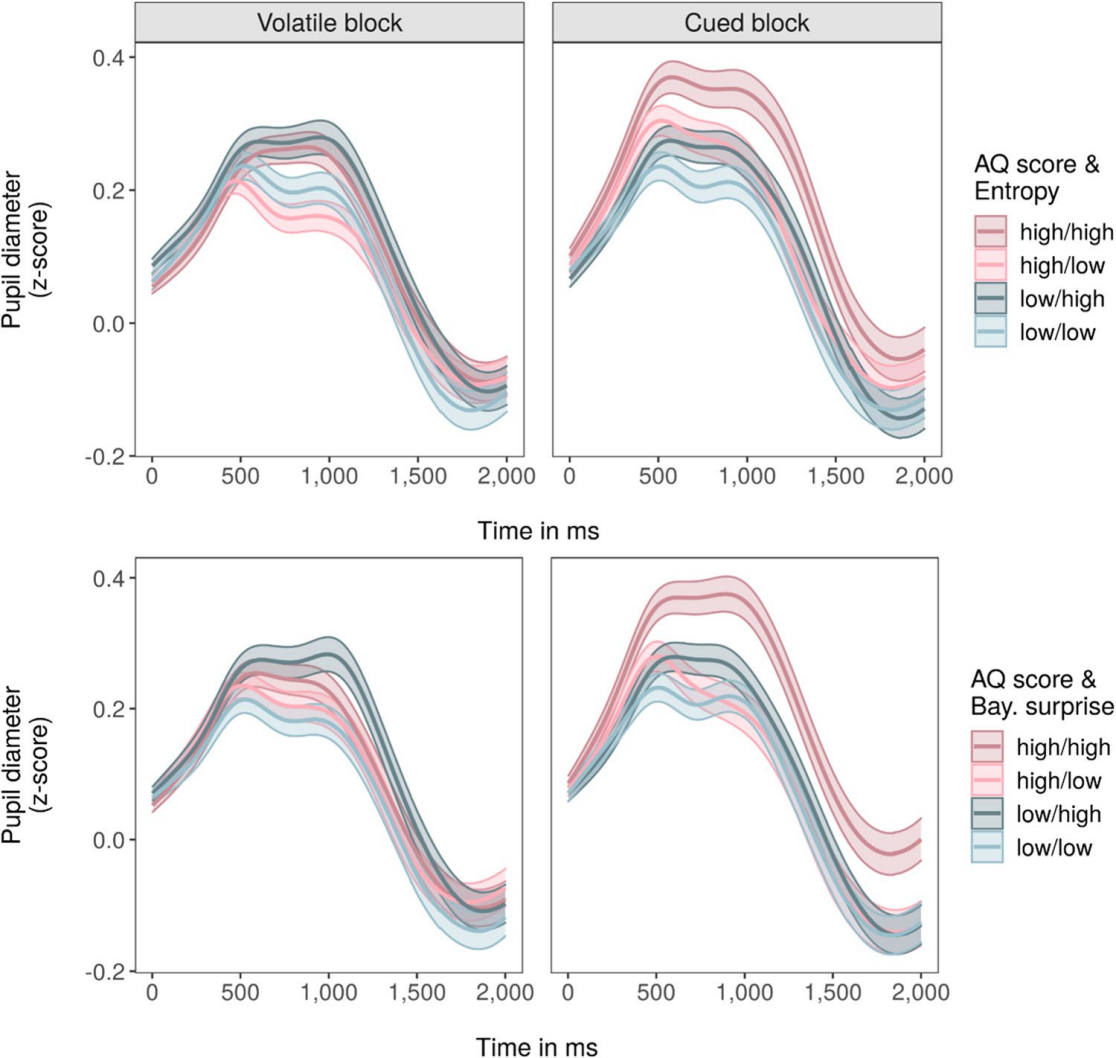
Pupil responses to choice uncertainty (entropy) and Bayesian surprise, moderated by AQ scores. *Notes*. Pupil responses during outcome presentation to choice uncertainty (entropy; top row) and Bayesian surprise (bottom row) are presented separately for the two task blocks (columns). Colors differentiate between responses for trials with high or low entropy/Bayesian surprise (defined as values within participant-specific upper and lower quartile) and participants scoring high or low on the AQ (defined as values above or below the sample-based median). These quartile- and median-based categorizations of high versus low entropy/Bayesian surprise trials and high versus low AQ scores, respectively, were not used in any of the statistical models and only applied here for illustration purposes. Reddish colors indicate a high, blueish colors a low AQ score, darker shades represent high, brighter shades low entropy/Bayesian surprise values. Mean (solid line) and standard error of the mean (shaded area) were calculated for each sample of the z-scored and baseline-corrected pupil signal during outcome presentation

Specifically, inclusion of AQ scores revealed a significant three-way interaction with Bayesian surprise and block (β = 0.05, *t* = 3.07, *p* < 0.01, [0.02, 0.08]), with pupil dilation scaling less with surprise in the volatile and more in the cued task block as individual AQ scores increased. This indicates a reduced differentiation between high and low surprise values in the volatile, and increased differentiation in the cued task block for individuals with higher AQ scores (Fig. 4; Supplementary Figure S3).

**Fig. 5:**
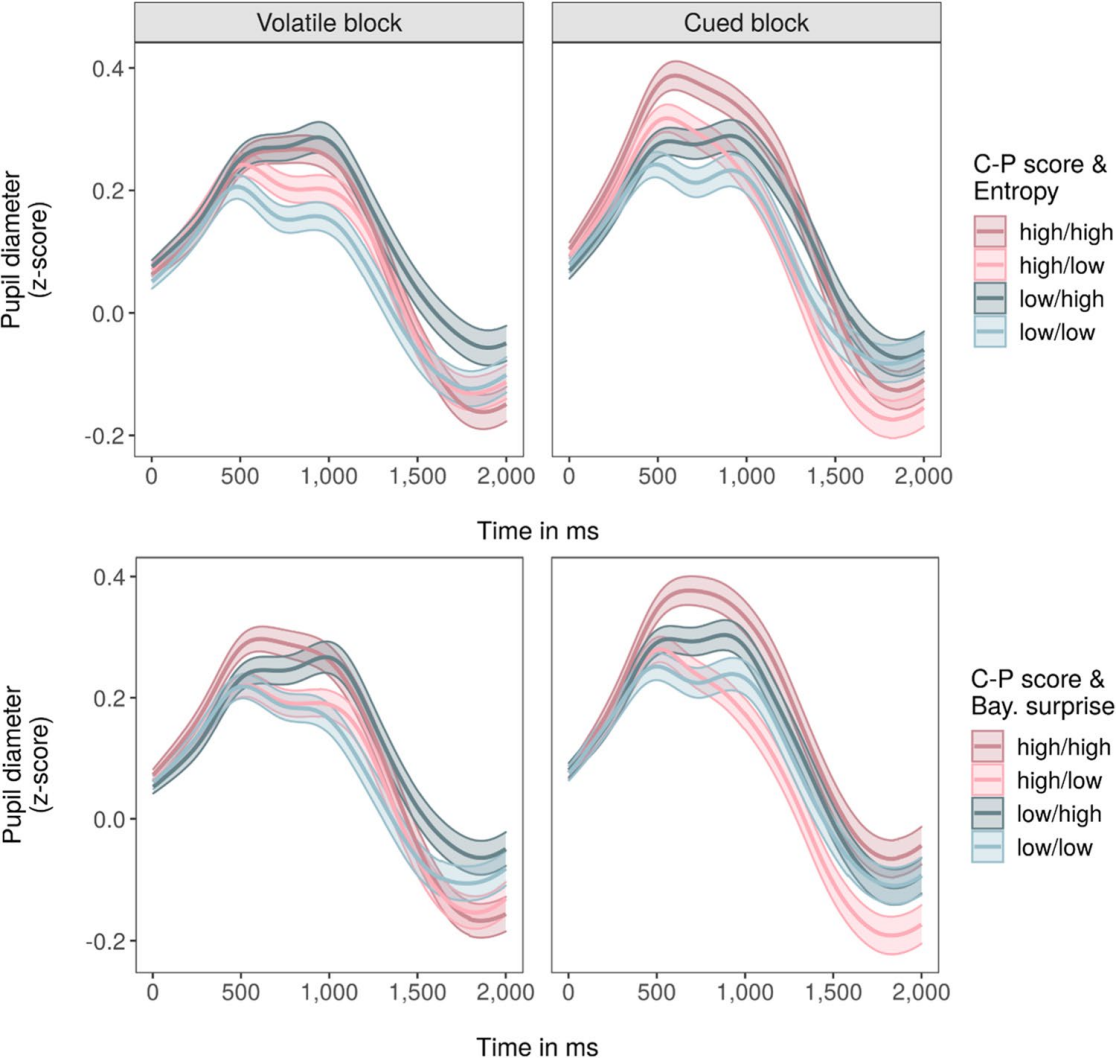
Pupil responses to choice uncertainty (entropy) and Bayesian surprise, moderated by CAPE-P scores (C-P). *Notes*. Pupil responses during outcome presentation to choice uncertainty (entropy; top row) and Bayesian surprise (bottom row) are presented separately for the two task blocks (columns). Colors differentiate between responses for trials with high or low entropy/Bayesian surprise (defined as values within participant-specific upper and lower quartile) and participants scoring high or low on the CAPE-P (C-P; defined as values above or below the sample-based median). These quartile- and median-based categorizations of high versus low entropy/Bayesian surprise trials and high versus low CAPE-P scores, respectively, were not used in any of the statistical models and only applied here for illustration purposes. Reddish colors indicate a high, blueish colors a low CAPE-P score, darker shades represent high, brighter shades low entropy/Bayesian surprise values. Mean (solid line) and standard error of the mean (shaded area) were calculated for each sample of the z-scored and baseline-corrected pupil signal during outcome presentation

In contrast, inclusion of CAPE-P scores (Table 3) yielded a significant three-way interaction with choice uncertainty and block (β = 0.04, *t* = 2.22, *p* = 0.03, [0.01, 0.07]), with pupil dilation scaling less with choice uncertainty in the volatile and more in the cued task block as individual CAPE-P scores increased. This indicates a reduced differentiation between high and low choice uncertainty values in the volatile, and increased differentiation in the cued task block for individuals with higher CAPE-P scores (Fig. 5; Supplementary Figure S4).

## Discussion

Using a probabilistic reversal learning task and concurrent pupillometry, this study investigated the association of autistic-like traits (AQ) and psychotic-like experiences (CAPE-P) with uncertainty processing in a sample of neurotypical adults. In contrast to findings from reversal learning studies using clinical samples (Culbreth et al., 2016; Deserno et al., 2020; Mussey et al., 2015; Waltz et al., 2013), the amount of switching per se was not significantly elevated in individuals with higher AQ or CAPE-P scores. This may be due to lower symptom load in neurotypical samples as well as differences in task design (e.g., regarding the use of monetary rewards [Culbreth et al., 2016; Deserno et al., 2020; Mussey et al., 2015; Waltz et al., 2013] or different risk conditions [Culbreth et al., 2016; Deserno et al., 2020; Waltz et al., 2013]). Participants switched more often between the two choice options on high-risk compared to low-risk trials, possibly resulting from increased uncertainty about the favorable response as well as “matching” behavior typical for probabilistic learning tasks (Feher da Silva et al., 2017). This effect was significantly smaller for participants with higher AQ scores, suggesting deficits in implicit probability learning (i.e., differentiating between risk conditions), in line with previous findings (Solomon et al., 2015).

Computational modelling revealed that subjective volatility was significantly higher for participants scoring high on CAPE-P in the cued, low-volatile task block, replicating prior findings of volatility overestimation in psychotic disorders such as schizophrenia (Cole et al., 2020; Deserno et al., 2020). This may seem surprising, as changes were announced in this block. It is possible that, given higher initial volatility assumptions, these participants still perceived the task states between announced changes as more unstable or that a failure to learn about the underlying risks and subsequently increased estimation uncertainty periodically caused more exploratory behavior, resulting in an increased volatility estimate. This uncertainty may have been too subtle to translate into behaviorally measured switching effects or overall differences in choice uncertainty.

Although the absence of AQ or CAPE-score correlations with subjective volatility during the volatile task block implies no effect of trait and experience scores on volatility representation under conditions of high volatility, pupillometric findings point to differences with regards to how individuals react to this volatility. Overall, pupil dilation scaled positively with both choice uncertainty and Bayesian surprise. While choice uncertainty expresses the uncertainty surrounding current beliefs of what may be the more favorable choice, Bayesian surprise signals the extent to which a belief should be updated. Hence, both relate to the informational value of the currently presented outcome for learning and belief updating: if uncertainty is high, the presented outcome may be particularly relevant to update beliefs and reduce uncertainty; if surprise is high, it indicates that a relatively larger belief update is warranted, possibly due to a change in the risk associated with different outcomes. Their associations with pupil dilation replicate previous findings (Hämmerer et al., 2019; Kreis et al., 2021) and fit well with the assumption that pupil size as an indicator for LC-NE activity signals neural gain and learning (Eldar et al., 2013; Joshi et al., 2016). Interestingly, the extent to which pupil dilation scaled with choice uncertainty and Bayesian surprise was moderated by questionnaire scores and task block. Individuals with higher AQ scores showed a diminished differentiation between high and low Bayesian surprise values in the volatile, and enhanced differentiation in the cued task block. Similarly, individuals with higher CAPE-P scores showed a diminished differentiation between high and low choice uncertainty values in the volatile, and enhanced differentiation in the cued task block. In theory, reduced pupil size adaptation in response to these learning signals in the volatile task block could be caused by an overestimation of volatility which renders all stimuli similarly surprising and worth directing one’s attention to. However, given that subjective volatility estimates in this block were not significantly related to any of the questionnaire scores, this seems unlikely. Instead, these results might be caused by a hypersensitivity to volatility which increases the difficulty of keeping track of underlying changes in uncertainty and surprise or decreases the subjective relevance of doing so. With risk conditions constantly changing, high-scoring and volatility-sensitive participants may have struggled to identify relevant learning signals or gave up tracking them. In contrast, the increased pupil size adaptation in response to choice uncertainty and Bayesian surprise in the cued task block may in fact be caused by an overestimation of volatility in an objectively rather stable environment, where belief updating in response to these learning signals should be less drastic than in volatile environments. When volatility is overestimated, high choice uncertainty and Bayesian surprise values may be falsely attributed to changes in underlying risk conditions when they in fact simply reflect the current and irreducible risk condition and task-inherent statistical deviations. The positive correlations between subjective volatility in the cued task block and CAPE-P as well as AQ scores (albeit not statistically significant for the latter), align with this interpretation.

These results fit well with previous findings of diminished pupil responses to surprise and uncertainty in individuals with autism (Lawson et al., 2017) and schizophrenia (Kreis et al., 2021). Diminished pupil responses may reflect aberrant norepinephrinergic (NE) signaling, which has been proposed to underlie altered uncertainty processing in both autism and schizophrenia (Kreis et al., 2021; Strauss et al., 2013; Van de Cruys et al., 2014). The locus coeruleus-NE system receives input from the anterior cingulate cortex, a brain region that, together with the insula and the prefrontal cortex, is critically implicated in decision-making under and processing of uncertainty. In both autism and schizophrenia, abnormal activity and connectivity of these regions may contribute to misestimation of uncertainty and altered decision-making in uncertain task environments (Fromm et al., 2022; Strauss et al., 2013; Van de Cruys et al., 2014). To what extent this is true for neurotypical individuals with elevated autistic- and psychotic-like traits and experiences remains to be unraveled, though similar activation alterations during learning under uncertainty have been observed in individuals with delusions (Fromm et al., 2022) and at risk for psychosis (Cole et al., 2020).

Notably, pupil responses to choice uncertainty (entropy) and Bayesian surprise were affected differently by the different traits and experiences. This divergence may indicate that choice uncertainty-representation related processes are more vulnerable to psychotic-like experiences and surprise-representation related processes more vulnerable to autistic-like traits, but this question warrants further investigation. Future studies should aim to include larger samples or preselect participants scoring particularly high and low on the AQ and the CAPE-P to include a wider range of trait and experience scores.

It should be noted that the model space in this study is not exhaustive, and that other models have provided reasonable computational mechanisms in similar task, such as the Hierarchical Gaussian Filter model (HGF) by Mathys et al. (2011) and the Ideal-Observer model by Behrens et al. (2007). The HGF, however, is more suitable for tasks that implement drifting risk changes, whereas risk remained fixed within each reversal period in the task used in this study. The Ideal-Observer model offers only a normative account, i.e., how individuals are supposed to behave under ideal circumstances, rather than an explanatory account which was more relevant to this study. In addition, the models employed here are deeply rooted within the Markov theory, and prior work has provided ample evidence regarding their efficiency and appropriateness (e.g., Hämmerer et al., 2019; Kreis et al., 2021; Schlagenhauf et al., 2014). Hence, the choice of model space in this study also enables direct replication in relation to prior work.

Block order was not counterbalanced in this study to maximize the difference between the cued and the volatile block and create a truly volatile and unpredictable experience in the volatile block. Timing of change points and order of the different risk conditions were identical in both blocks to ensure they only differed in unpredictability, i.e., volatility, of risk changes. If the cued block were to be administered first, participants may learn about the timing of change points based on the change announcements and transfer that knowledge to the subsequent volatile block, where hidden changes consequentially might be easier to detect. This would reduce the experienced volatility in this block and would diminish the difference between both blocks, with both being “cued” to some degree. Nevertheless, investigating this effect may be interesting to address questions above and beyond those of the current study, for example, to what extent the ability to transfer this acquired knowledge about change points to decision-making in the volatile block varies with autism- or psychosis-like traits and experiences. Notably, some research indicates increased subjective volatility under volatile conditions even if following stable conditions (Browning et al., 2015), and recent studies have demonstrated that starting with an easy block, where changes in risk are less difficult to identify, can increase susceptibility to subsequent volatility because stronger expectations have been formed (Reed et al., 2020; Sheffield et al., 2022; Suthaharan et al., 2021). However, task designs differed with either no risk changes appearing under stable conditions at all (Browning et al., 2015), or varying risk conditions between blocks (Reed et al., 2020; Sheffield et al., 2022; Suthaharan et al., 2021). In contrast, and due to reasons outlined above, the task used in the current study may be more vulnerable to such order manipulations.

Together, these results provide important insights into how autistic- and psychotic-like traits and experiences are related to processing and representation of different kinds of uncertainty – even in neurotypical individuals. While psychotic-like experiences were associated with overestimation of volatility in a low-volatile period of the task, behavioral results further point to a link between autistic-like traits and risk misestimation. Psychophysiological results revealed a distinct pattern of abnormal neural gain adaptation to uncertainty and surprise for psychotic- and autistic-like traits and experiences, respectively. This is in line with theoretical accounts of abnormal uncertainty processing and consequentially aberrant belief updating in psychosis and autism spectrum disorders.

## Supplementary Information

ESM 1 (PDF 1059 kb)

## Footnotes

1 We acknowledge that “risk” is assumed to be a perfectly known entity in behavioral economics (e.g., Johnson & Busemeyer, 2010) but treat it as statistical uncertainty in the context of this study so that it does not confuse with the estimation uncertainty that we also measure.
