# Supplementary material for: Aberrant uncertainty processing is linked to psychotic-like experiences, autistic traits, and is reflected in pupil dilation during probabilistic learning
Source: Cogn Affect Behav Neurosci. 2023 Mar 28;23(3):905–19. doi: 10.3758/s13415-023-01088-2 (PMC10390366; doi:10.3758/s13415-023-01088-2)
Supplement: Supplementary file 1 — (PDF 1059 kb) [file 13415_2023_1088_MOESM1_ESM.pdf]

Supplementary Information for:

Aberrant Uncertainty Processing Is Linked to Psychotic-like Experiences, Autistic Traits and Reflected in Pupil Dilation During Probabilistic Learning

Isabel Kreis

Department of Psychology, UiT – The Arctic University of Norway, Tromsø, Norway

NORMENT, Institute of Clinical Medicine, University of Oslo, Oslo, Norway

Lei Zhang

Department of Psychology, UiT – The Arctic University of Norway

Social, Cognitive and Affective Neuroscience Unit, Department of Cognition, Emotion, and Methods in Psychology, Faculty of Psychology, University of Vienna, Vienna, Austria

Institute for Systems Neuroscience, University Medical Center Hamburg-Eppendorf, Hamburg, Germany

Centre for Human Brain Health, School of Psychology, University of Birmingham, Birmingham, United Kingdom

Institute for Mental Health, School of Psychology, University of Birmingham, Birmingham, United Kingdom

Matthias Mittner

Department of Psychology, UiT – The Arctic University of Norway, Tromsø, Norway

Leonard Syla

Department of Psychology, UiT – The Arctic University of Norway, Tromsø, Norway

Claus Lamm

Social, Cognitive and Affective Neuroscience Unit, Department of Cognition, Emotion, and Methods in Psychology, Faculty of Psychology, University of Vienna, Vienna, Austria

Vienna Cognitive Science Hub, University of Vienna, Vienna, Austria

Gerit Pfuhl

Department of Psychology, UiT – The Arctic University of Norway, Tromsø, Norway

Department of Psychology, Norwegian University of Science and Technology, Trondheim, Norway

Supplementary Methods: Working Memory Task

Working memory capacity was assessed with the visual digit span and the matrix span task of a computerized open source working memory test battery (Stone & Towse, 2015). On each trial of the digit span task, participants were presented with several digits between 10 and 99, one after another, and had to recall them in the correct order. The number of digits to recall (i.e. task load) increased by one after every third trial, ranging from two to seven. The maximum number of digits recalled in the correct order was used as an index for working memory capacity in the verbal-numerical domain. Load conditions and general structure were identical for the matrix span task, where instead of digits, indicated grid locations in a 4x4 grid had to be recalled. Here, maximum number of locations recalled in the correct order was used as an index for working memory capacity in the visual-spatial domain.

Supplementary Methods: Computational Models

Computation models were fitted to participants' choice data for the cued and the volatile block of the prediction task, respectively. The models included a simple win-stay-loose-shift model (WSLS), four different Reinforcement Learning models, and two variants of a Hidden Markov Model.

All models are based on relating participant's choices with their subsequent outcome in one way or another. For the prediction task, those choices describe the prediction of either the left or the right tilted Gabor patch on a given trial. The outcome was then simply the observation of either the left or the right tilted patch, following the prediction. A positive feedback was defined as a match between prediction and outcome and assigned the reward value +1. A negative feedback was in turn a mismatch between

prediction and outcome and assigned the 'reward' value -1 (also referred to as 'punishment').

Choice of models

The WSLS model (Worthy & Todd Maddox, 2014) assumes that participants continue to choose one of the two choice options (left/right) if they were rewarded for this choice on the previous trial but shift to the other option when they were not. It was included to allow for the fact that participants might employ a very simplistic prediction strategy, basing their choices purely on the outcome of the previous trial. The four RL models assume a more long-term integration of prediction errors and prediction values of the two choice options. The four versions differed in how this information was integrated. In the simplest Rescorla-Wagner model (RL_{RW}; Rescorla & Wagner, 1972), values for only the chosen action (i.e. predict the left or the right-tilted Gabor patch) were updated via learning rate weighted prediction errors. The reward-punishment model (RL_{RP}; den Ouden et al., 2013) introduces different learning rates for positive (prediction correct) vs. negative feedback (prediction incorrect), since participants might respond differently to those events. Given the anti-correlated task structure, participants might learn about the values of the two choice options concurrently. This was accounted for in a counterfactual updating model (RL_{CF}; Gläscher et al., 2008), where values for both actions were updated simultaneously. In an additional model based on Pearce and Hall (RL_{PH}; Pearce & Hall, 1980), the learning rate was adaptive and varied across the course of the respective task block. This could capture the fact that the weight assigned to a prediction error, and thus the extent of value updating, might change throughout the task and be larger after change points. The Hidden Markov Models (HMM; Schlagenhauf et al., 2014) were included to allow for a more strategic decision-making process that rests on a higher-level representation

of the different task-states. They assume that participants choose between 'left' or 'right' based on their belief of being in a state of the task where either the left- or the right-tilted Gabor patch is more common, and on their estimated probability for the two states to change. To account for the fact that participants may be more or less sensitive to positive (prediction correct) vs. negative feedback (prediction incorrect), one HMM variant allowed for the effects of those outcomes to differ (HMM_{RP}), and one assumed identical effects of positive and negative feedback (HMM).

For the cued task block, additional variants of all models were specified that implemented a reset of beliefs about choice values and current state probabilities at each time point where participants were informed about a change in the underlying stimulus distribution. It was expected that after each of these announced change points, beliefs would be reset to their initial values (0.5). All models are described in detail below (I – V), with a summary of the models' free parameters and results of model comparisons listed in Table S1 and S2.

(I) Win-Stay-Loose-Shift model

As the first and simplest model, a Win-Stay-Lose-Shift model was fitted (WSLS; Worthy & Todd Maddox, 2014). This model assumes that participants continue to choose one of the two available options (left/right) if they were rewarded for this choice on the previous trial ('win-stay'; i.e. their prediction turned out to be correct). Likewise, participants are assumed to pick the other option if they were not rewarded ('lose-shift'; i.e. their prediction turned out to be incorrect). Let A and B be the two actions, the value of 'staying' with the same choice option after a 'win' (meaning the net gain on the current trial is equal to or greater than zero) is then calculated as:

$$V(A|A, win) = 1 \quad (1)$$

$$V(B|A, win) = -1 \quad (2)$$

Similarly, the value of 'switching' to the other option after a 'loss' (meaning the net gain on the current trial is less than zero) is:

$$V(B|A, loss) = 1 \quad (3)$$

$$V(A|A, loss) = -1 \quad (4)$$

(II) Standard Rescorla-Wagner model

The second model was a standard Rescorla-Wagner reinforcement learning (RL_{RW}; Rescorla & Wagner, 1972) model with a constant learning rate and value updates for only the choses action. The updating rule of this model defines that for each trial t, the value V of the current choice depends on the value and the prediction error δ (the difference between reward and expected value) of the previous trial $t-1$, weighted by the learning rate α:

$$V_t = V_{t-1} + \alpha \times \delta \quad (5)$$

Here, δ is the prediction error, calculated as:

$$\delta = (R_{t-1} - V_{t-1}) \quad (6)$$

To account for the possibility that the effects of 'rewards' (i.e. correct predictions) and 'punishments' (i.e. incorrect predictions) on learning might differ, an variant of the model was fitted with separate learning rates α for rewards and punishments (model: RL_{RP}; den Ouden et al., 2013).

(III) Counterfactual Reinforcement Learning model

The third model was a counterfactual updating model (RL_{CF}; Gläscher et al., 2008) that only differed from (II) in the way that values of both the chosen and the unchosen options were updated in parallel. The formula for the value update was the same as above but for the unchosen option (uc), a fictitious prediction error was used:

$$\delta^{f} = (-R_{t-1} - V_{t-1}^{uc}) \quad (7)$$

(IV) Pearce-Hall model

The fourth model was a Pearce-hall model with a dynamic learning rate (RL_{PH}; Pearce & Hall, 1980). Value update was similar to the standard RL_{RW} model (see II), with learning rate α weighted by an additional parameter, k, determining the effect of learning rate on value updates:

$$V_t = V_{t-1} + k \times \alpha_{t-1} \times (R_t - V_{t-1}) \quad (8)$$

Furthermore, learning rate α varied across trials, updated from the previous prediction error:

$$\alpha_t = \eta \times |(R_{t-1} - V_{t-1})| + (1 - \eta) \times \alpha_{t-1} \quad (9)$$

Here, η is the weighting factor of the prediction error when updating α_t on a given trial.

(V) Hidden Markov Model

Lastly, behavior was modelled with a Hidden Markov Model (HMM; Schlagenhauf et al., 2014). Here, it is assumed that participants make their choices (i.e. predict either the left or the right Gabor patch) based on their belief about the current state of the task as there being either predominantly left ('state L') or predominantly right ('state R') tilted patches.

While inferring the belief distribution over the different states, participants are assumed to treat action-reward pairs (i.e. the combination of their prediction and the 'reward' in terms of the prediction being correct or incorrect) as observations: $O_t = \{a_t, r_t\}$. A hidden state variable S_t represents a participant's estimation of such an action-outcome pair. The prior over the current state $P(S_t|S_{t-1})$ is calculated as the posterior belief from the previous trial modulated by the reversal probability γ (i.e., the transition probabilities between latent states) in a transition matrix:

$$P(S_t|S_{t-1}) = \begin{pmatrix} 1-\gamma & \gamma \\ \gamma & 1-\gamma \end{pmatrix} \tag{10}$$

Here, γ is a free parameter between 0 and 1.

The probability of observing the outcome corresponding to a given latent state depends on the probability c with which a reward (i.e. positive feedback indicating a correct prediction) indicates that the true latent state indeed corresponds to the choice made and the probability d with which a 'punishment' (i.e. negative feedback indicating an incorrect prediction) indicates that the latent state is *not* the one chosen by action a. It is updated as:

$$P(O_t|S_t) = 0.5 \times \begin{pmatrix} c & 1-c \\ 1-d & d \end{pmatrix} \tag{11}$$

Reward sensitivity c and punishment sensitivity d were treated as free parameters and initialized to lie between 0.5 and 1. As in the RL_{RP} model, this differentiation between 'rewards' and 'punishments' allowed for different effects of both in terms of their informativeness for model updating. This version of the model is here referred to as HMM_{RP}. For comparison, an additional version of the model was fitted where rewards and punishments were treated equally, with $c = d$. That version of the model is denoted as HMM.

On a given trial, the prior probability of S_t before any outcome has been observed is calculated from the state transition probabilities (see above) and the posterior probability of S_{t-1}:

$$P(S_t) = \sum_{S_{t-1}} P(S_t|S_{t-1})P(S_{t-1}) \tag{12}$$

After outcome observation, the posterior probability of S_t is then updated based on the prior $P(S_t)$ and the observed outcome O_t:

$$P(S_t) = \frac{P(O_t|S_t)P(S_t)}{\sum_{S_t} P(O_t|S_t)P(S_t)} \tag{13}$$

Softmax action selection

For models (I) – (IV), option values were translated into choice probabilities for options L and R with a softmax action selection function:

$$p(R) = \frac{1}{1 - \mathrm{e}^{\beta\times(-(V_R - V_L)}}, \qquad p(L) = 1 - p(R) \tag{14}$$

Here, β is the inverse temperature, which determines the slope of the sigmoid function and the stochasticity (randomness) of the choices. The softmax function also ensures that the converted choice probabilities of both options would sum up to one. For the HMM models (V), state probabilities, rather than choice values, were directly used to represent choice probabilities without using the softmax function. This was also to reduce non-identifiable parameter estimations.

Model comparison

All model parameters were estimated using Hierarchical Bayesian Analysis (HBA; Gelman et al., 2013) implemented in the Stan language in R (Stan Development Team, 2016), adopted from the hBayesDM package (Ahn et al., 2017). For both task blocks separately, models were compared regarding their goodness-of-fit to explain the observed data whilst accounting for

model complexity. Model fit was compared using leave-one-out cross validation, calculated using the log-likelihood evaluated at the posterior simulations. Model fit was reported as leave-one-out information criterion (LOOIC), with lower LOOIC indicating better model fit. Model fit results are reported in Table S1 for the first, volatile block of the task and in Table S2 for the second, cued block of the task. The HMM_{RP} provided the best fit for both the volatile and the cued task block. Notably, our group recently conducted a study using the same task paradigm and an identical set of models. Here, a model recovery analysis revealed that all candidate models could be properly identified and recovered (Kreis et al., 2021).

Table S1

Task block 1 (volatile): Model fit and individual-level parameters

Model	LOOIC	no. of parameters	parameters
WSLS	9950	1	Inverse temperature: β
RL_{RW}	9761	2	Learning rate: α<br>Inverse temperature: β
RL_{CF}	9505	2	Learning rate: α<br>Inverse temperature: β
RL_{RP}	9717	3	Reward learning rate : α^{rew}<br>Punishment learning rate : α^{pun}<br>Inverse temperature: β
RL_{PH}	9756	4	Learning rate: α<br>Weighting factor prediction error: η<br>Weighting factor learning rate: *k*<br>Inverse temperature: β
HMM	9559	2	Transition probability: γ<br>Reward/punishment sensitivity: *c* (with $d = c$)
**HMM_{RP}**	**9391**	**3**	Transition probability: γ<br>Reward sensitivity: *c*<br>Punishment sensitivity: *d*

Notes: For each individual-level parameter, two group-level parameters were estimated, representing mean and standard deviation of the individual-level parameters, respectively.

Table S2

Task block 2 (cued): Model fit and individual-level parameters

Model	LOOIC	no. of parameters	parameters
WSLS	9982	1	Inverse temperature: β
RL_{RW}	9986	2	Learning rate: α<br>Inverse temperature: β
RL_{RW_reset}	9189	2	Learning rate: α<br>Inverse temperature: β
RL_{CF}	10248	2	Learning rate: α<br>Inverse temperature: β
RL_{CF_reset}	8897	2	Learning rate: α<br>Inverse temperature: β
RL_{RP}	10222	3	Reward learning rate : α^{rew}<br>Punishment learning rate : α^{pun}<br>Inverse temperature: β
RL_{RP_reset}	8796	3	Reward learning rate : α^{rew}<br>Punishment learning rate : α^{pun}<br>Inverse temperature: β
RL_{PH}	9981	4	Learning rate: α<br>Weighting factor prediction error: η<br>Weighting factor learning rate: k<br>Inverse temperature: β
RL_{PH_reset}	9189	4	Learning rate: α<br>Weighting factor prediction error: η<br>Weighting factor learning rate: k<br>Inverse temperature: β
HMM	9448	2	Transition probability: γ<br>Reward/punishment sensitivity: c (with $d = c$)
$HMM_{\_reset}$	8902	2	Transition probability: γ<br>Reward/punishment sensitivity: c (with $d = c$)
HMM_{RP}	9018	3	Transition probability: γ<br>Reward sensitivity: c<br>Punishment sensitivity: d
**HMM_{RP_reset}**	**8416**	**3**	Transition probability: γ<br>Reward sensitivity: c<br>Punishment sensitivity: d

Notes: For each individual-level parameter, two group-level parameters were estimated, representing mean and standard deviation of the individual-level parameters, respectively.

Supplementary Results

Questionnaire scores (AQ and CAPE-P) are not related to demographic or working memory variables

Questionnaire scores were not related to age (AQ: $\rho = -0.13$, $p = .35$; CAPE-P: $\rho = -0.22$, $p = .11$), education (AQ: $\chi^2(2) = 0.58$, $p = .75$; CAPE-P: $\chi^2(2) = 0.73$, $p = .69$), gender (AQ: W = 294.50, $p = 0.57$; CAPE-P: W = 310.50, $p = .79$), verbal-numerical (AQ: $\rho = 0.03$, $p = .85$; CAPE-P: $\rho = 0.04$, $p = .78$) or visual-spatial working memory capacity (AQ: $\rho = 0.03$, $p = .81$; CAPE-P: $\rho = 0.22$, $p = .12$).

Choice Uncertainty (Entropy) and Bayesian surprise differ by task conditions but not questionnaire scores

In two separate linear mixed-effects models, both choice uncertainty (entropy) and Bayesian surprise were significantly higher on high-risk trials (Table S3). For choice uncertainty, this effect was more pronounced within the cued task block (block*risk: $\beta = 0.18$, $t = 2.95$, $p < .01$, 95% CI [0.06, 0.31]), where high- and low-risk conditions would be easier to distinguish due to the absence of hidden changes. Bayesian surprise was lower in the cued task block, indicating reduced belief updating under low volatility ($\beta = -0.40$, $t = -4.53$, $p < .001$, [-0.58, -0.23]).

Table S3

Linear mixed-effects model results for latent HMM_{RP} variables by task conditions

DV		β	*t*	*p*	CI (95%)	R^2_M	R^2_C
Entropy						0.03	0.42
	Block	-0.07	-0.66	.51	[-0.28, 0.14]		
	Risk	0.23	5.18	<.001	[0.14, 0.32]		
	Block*Risk	0.18	2.95	<.01	[0.06, 0.31]		
Bayesian surprise						0.05	0.38
	Block	-0.40	-4.53	<.001	[-0.58, -0.23]		
	Risk	0.20	6.75	<.001	[0.14, 0.26]		
	Block*Risk	0.01	0.12	.90	[-0.8, 0.09]		

Notes: Coefficients of the fixed effects in the linear mixed-effects models for the different dependent variables (DV); Entropy = choice uncertainty (HMM_{RP}); Bayesian surprise = cube root transformed Bayesian surprise (HMM_{RP}); Block = contrast of the second, cued task block to the first, volatile task block; Risk = contrast of the high- to the low-risk condition;; R^2m = marginal R^2, i.e. proportion of variance explained by the fixed effects alone; R^2c = conditional R^2, i.e. proportion of variance explained by both the fixed and random effects (R^2m and R^2c based on Nakagawa & Schielzeth, 2013). Results are rounded to two decimal places.

Inclusion of AQ and CAPE-P scores, respectively, did not reveal any significant score-related main or interaction effects – neither in the choice uncertainty model (***AQ-model:*** AQ: β = 0.06, *t* = 0.65, *p* = .52, 95% CI [-0.12, 0.23]; block*AQ: β = 0.06, *t* = 0.54, *p* = .59, [-0.15, 0.27]; risk*AQ: β = -0.02, *t* = -0.39, *p* = .69, [-0.10, 0.07]; block*risk*AQ: β = 0.02, *t* = 0.29, *p* = .77, [-0.11, 0.14]; ***CAPE-P model:*** CAPE-P: β = -0.05, *t* = -0.53, *p* = .60, [-0.22, 0.13]; block*CAPE-P: β = 0.15, *t* = 1.46, *p* = .15, [-0.06, 0.36]; risk*CAPE-P: β = -0.03, *t* = -0.80, *p* = .43, [-0.12, 0.05]; block*risk*CAPE-P: β = 0.01, *t* = -0.12, *p* = .91, [-0.13, 0.12]), nor the Bayesian surprise model (***AQ-model:*** AQ: β = 0.05, *t* = 0.60, *p* = .55, [-0.11, 0.21]; block*AQ: β = 0.02, *t* = 0.27, *p* = .79, [-0.16, 0.20]; risk*AQ: β = -0.02, *t* = -0.81, *p* = .42, [-0.08, 0.04]; block*risk*AQ: β = 0.01, *t* = 0.23, *p* = .82, [-0.07, 0.09]; ***CAPE-P-model:*** CAPE-P: β = 0.13, *t* = 1.63, *p* = .11, [-0.03, 0.29]; block*CAPE-

P: β = 0.02, t = 0.24, p = .81, [-0.16, 0.20]; risk*CAPE-P: β = -0.01, t = -0.36, p = .72, [-0.07, 0.05]; block*risk*CAPE-P: β = 0.00, t = 0.08, p = .94, [-0.08, 0.09]).

Supplementary Figures: Accuracy by questionnaire scores

Figure S1

Relationship Between Trait and Experience Scores and Proportion of Accurate Predictions

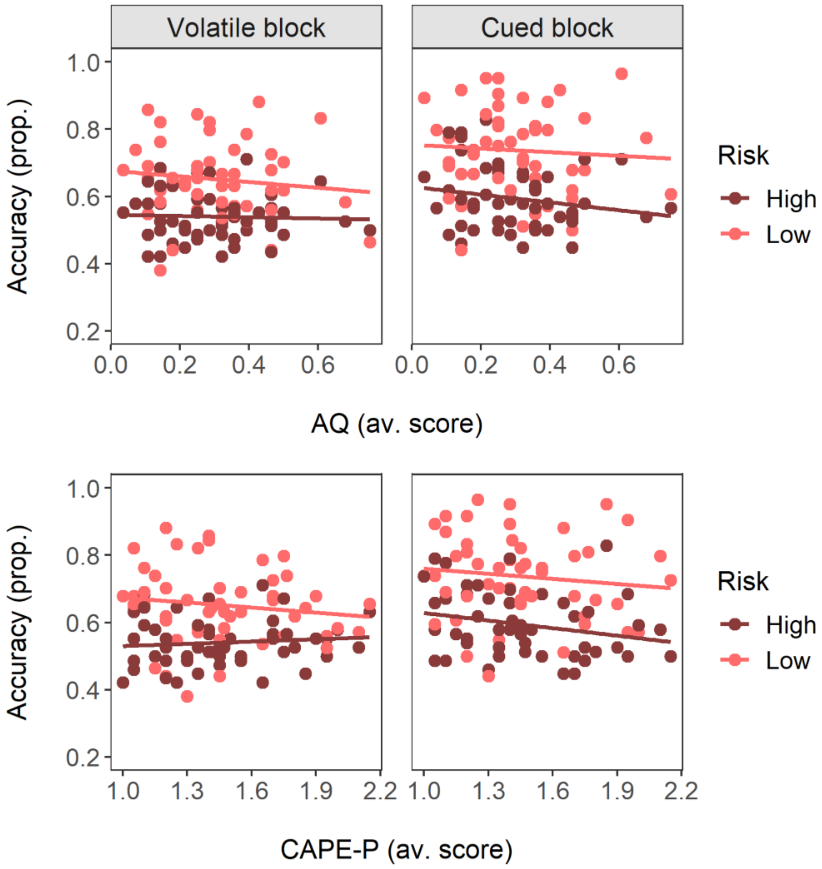

Notes. Proportion of accurate predictions is presented separately for the different task blocks (columns) and risk conditions (color). Trait and experience scores are average scores of AQ (top row) and CAPE-P (bottom row). Points represent values per participant and task condition; lines are regression lines (linear model) to demonstrate trends.

Supplementary Figures: Interaction Effects

Figures below display predicted values of the 3-way interaction terms from the linear-mixed effects models of pupil size by task block, choice uncertainty/Bayesian surprise, and AQ/CAPE-P scores, respectively, plotted with the sjPlot package (version 2.8.9; Lüdecke, 2021). In each Figure, the continuous questionnaire score variables were grouped by minimum (blue color), median (purple color), and maximum value (red color) for demonstration purposes. Left panels present values for the first, volatile task block, and right panels values for the second, cued task block.

Figure S2

Predicted Values of Maximum Pupil Dilation by Block, Choice Uncertainty (Entropy), and AQ Scores

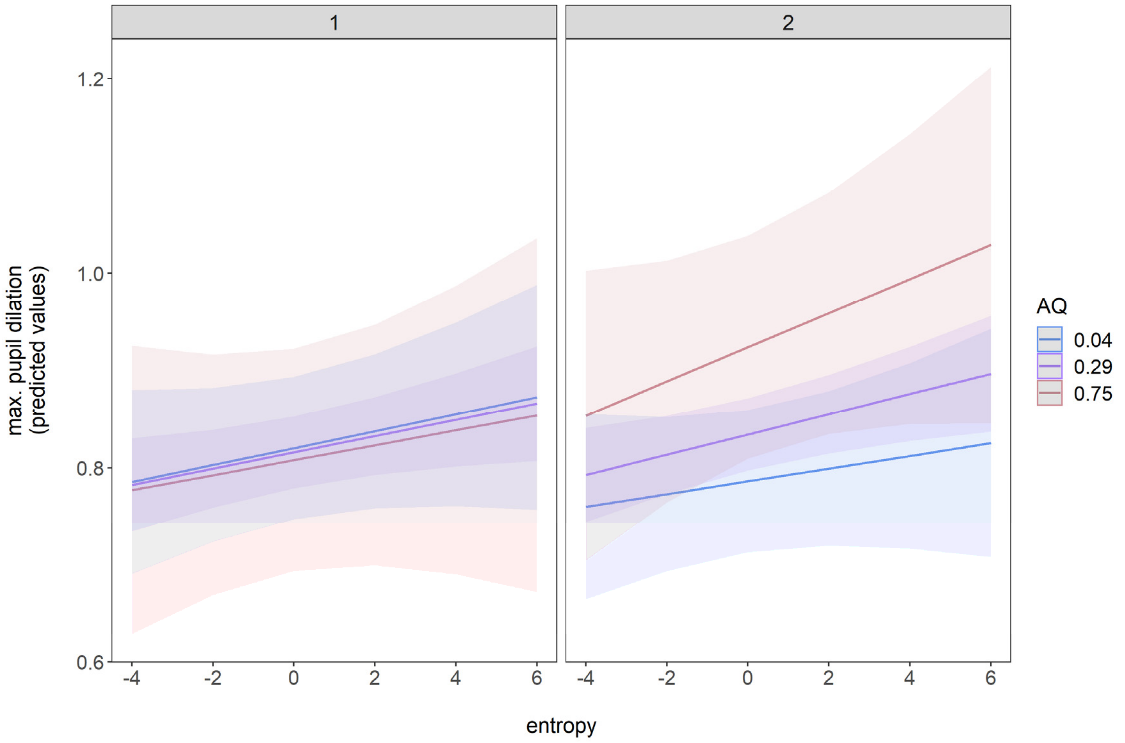

Figure S3

Predicted Values of Maximum Pupil Dilation by Block, Bayesian Surprise, and AQ Scores.

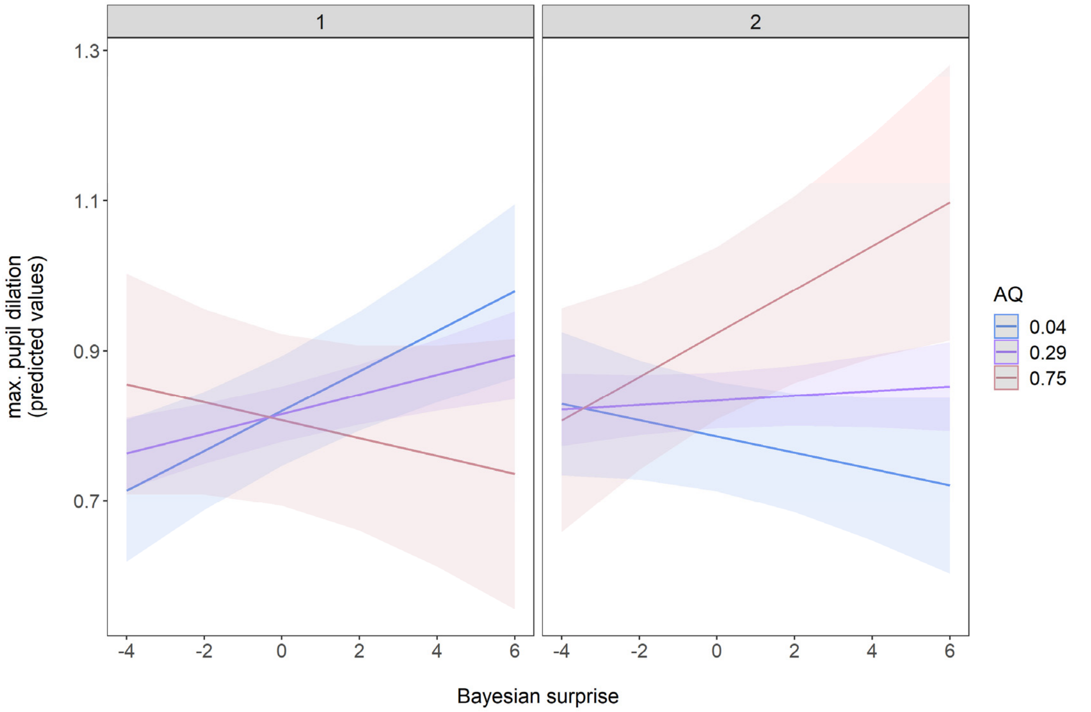

Figure S4

Predicted Values of Maximum Pupil Dilation by Block, Choice Uncertainty (Entropy), and CAPE-P Scores

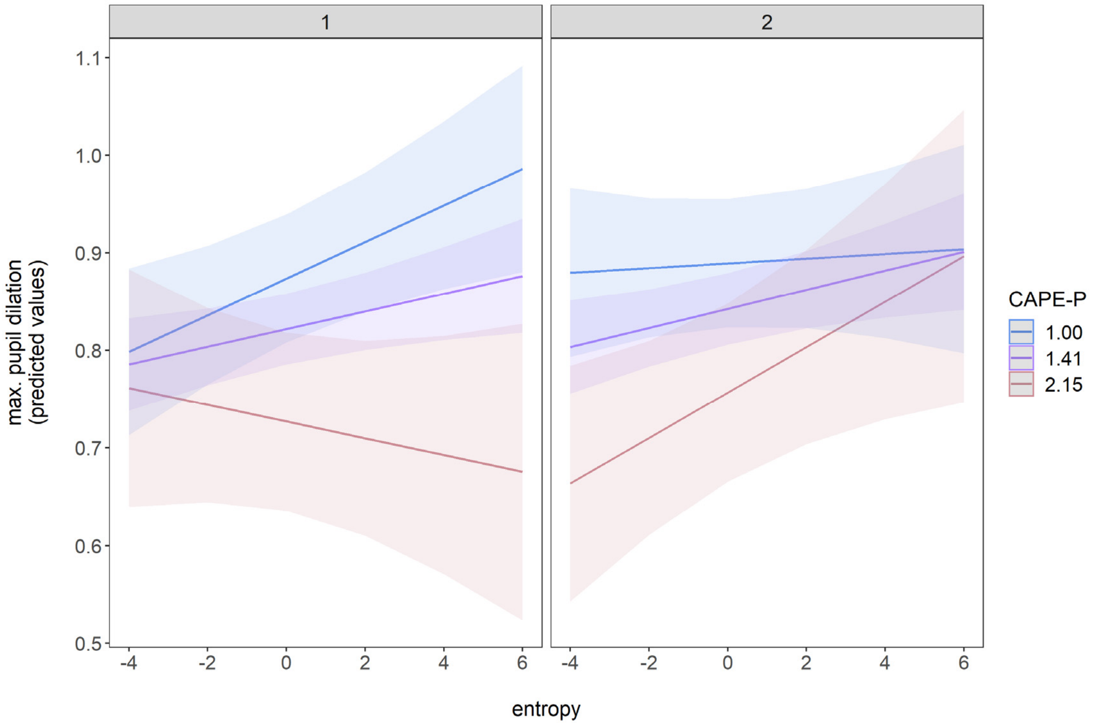

Figure S5

Predicted Values of Maximum Pupil Dilation By block, Bayesian Surprise, and CAPE-P Scores

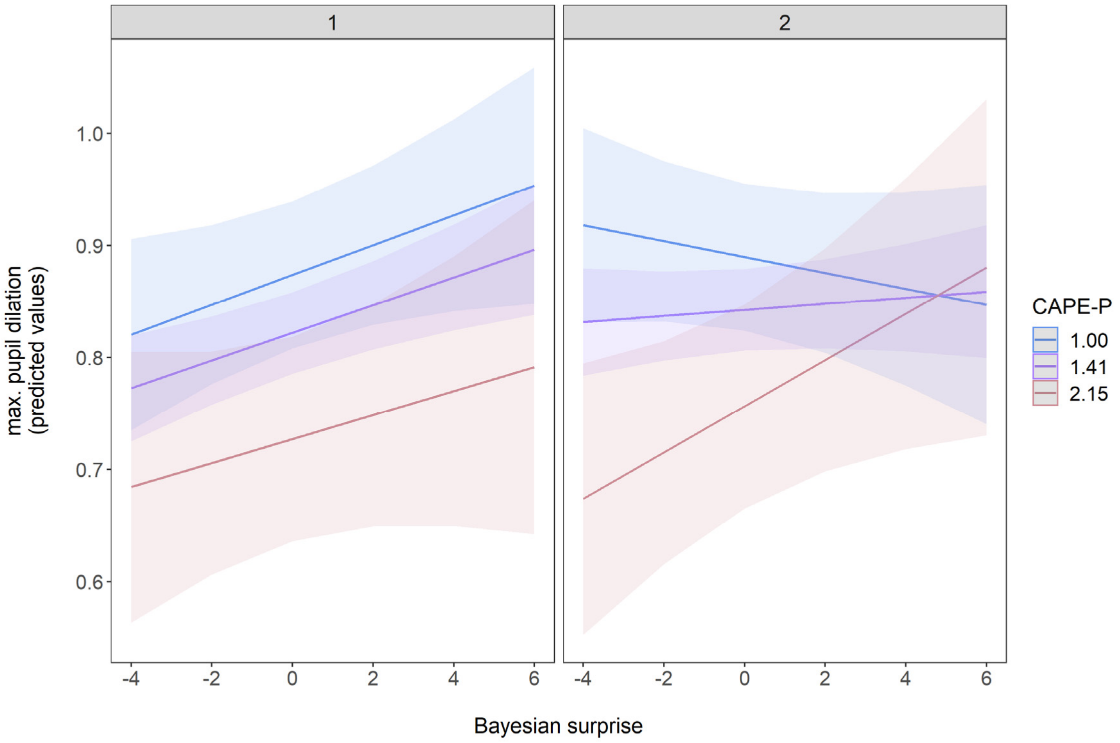

References (Supplementary Information):

Ahn, W. Y., Haines, N., & Zhang, L. (2017). Revealing Neurocomputational Mechanisms of Reinforcement Learning and Decision-Making With the hBayesDM Package. *Comput Psychiatr*, *1*, 24-57. https://doi.org/10.1162/CPSY_a_00002

den Ouden, Hanneke E. M., Daw, Nathaniel D., Fernandez, G., Elshout, Joris A., Rijpkema, M., Hoogman, M., . . . Cools, R. (2013). Dissociable Effects of Dopamine and Serotonin on Reversal Learning. *Neuron*, *80*(4), 1090-1100. https://doi.org/https://doi.org/10.1016/j.neuron.2013.08.030

Gelman, A., Carlin, J. B., Stern, H. S., Dunson, D. B., Vehtari, A., & Rubin, D. B. (2013). *Bayesian data analysis*. CRC press.

Gläscher, J., Hampton, A. N., & O'Doherty, J. P. (2008). Determining a Role for Ventromedial Prefrontal Cortex in Encoding Action-Based Value Signals During Reward-Related Decision Making. *Cerebral Cortex*, *19*(2), 483-495. https://doi.org/10.1093/cercor/bhn098

Kreis, I., Zhang, L., Moritz, S., & Pfuhl, G. (2021). Spared performance but increased uncertainty in schizophrenia: Evidence from a probabilistic decision-making task. *Schizophrenia Research*. https://doi.org/https://doi.org/10.1016/j.schres.2021.06.038

Lüdecke, D. (2021). *sjPlot: Data Visualization for Statistics in Social Science,* R package version 2.8.9. https://CRAN.R-project.org/package=sjPlot

Nakagawa, S., & Schielzeth, H. (2013). A general and simple method for obtaining R2 from generalized linear mixed-effects models. *Methods in Ecology and Evolution*, *4*(2), 133-142. https://doi.org/10.1111/j.2041-210x.2012.00261.x

Pearce, J. M., & Hall, G. (1980). A model for Pavlovian learning: Variations in the effectiveness of conditioned but not of unconditioned stimuli. *Psychological Review*, *87*(6), 532-552. https://doi.org/10.1037/0033-295X.87.6.532

Rescorla, R. A., & Wagner, A. R. (1972). A theory of Pavlovian conditioning: Variations in the effectiveness of reinforcement and nonreinforcement. In A. H. Black & W. F. Prokasy (Eds.), *Classical conditioning II: Current research and theory* (pp. 64–99). Appleton Century Crofts.

Schlagenhauf, F., Huys, Q. J. M., Deserno, L., Rapp, M. A., Beck, A., Heinze, H.-J., . . . Heinz, A. (2014). Striatal dysfunction during reversal learning in unmedicated schizophrenia patients. *NeuroImage*, *89*, 171-180. https://doi.org/https://doi.org/10.1016/j.neuroimage.2013.11.034

Stan Development Team. (2016). *RStan: the R interface to Stan*. R package version, 2.16.2. https://doi.org/http://mc-stan.org/

Stone, J. M., & Towse, J. N. (2015). A working memory test battery: Java-based collection of seven working memory tasks. *Journal of Open Research Software*, *3*(1), e5. https://doi.org/http://doi.org/10.5334/jors.br

Worthy, D. A., & Todd Maddox, W. (2014). A comparison model of reinforcement-learning and win-stay-lose-shift decision-making processes: A tribute to W.K. Estes. *Journal of Mathematical Psychology*, *59*, 41-49. https://doi.org/https://doi.org/10.1016/j.jmp.2013.10.001
